# Aerosol jet printing of surface acoustic wave microfluidic devices

**DOI:** 10.1038/s41378-023-00606-z

**Published:** 2024-01-01

**Authors:** Joseph Rich, Brian Cole, Teng Li, Brandon Lu, Hanyu Fu, Brittany N. Smith, Jianping Xia, Shujie Yang, Ruoyu Zhong, James L. Doherty, Kanji Kaneko, Hiroaki Suzuki, Zhenhua Tian, Aaron D. Franklin, Tony Jun Huang

**Affiliations:** 1 Department of Biomedical Engineering, Duke University, Durham, NC 27708 USA; 2 Department of Electrical and Computer Engineering, Duke University, Durham, NC 27708 USA; 3 Department of Mechanical Engineering, Virginia Polytechnic Institute and State University, Blacksburg, VA 24061 USA; 4 Thomas Lord Department of Mechanical Engineering and Materials Science, Duke University, Durham, NC 27708 USA; 5 Deptartment of Precision Mechanics, Faculty of Science and Engineering, Chuo University, Tokyo, 112-8551 Japan; 6 Department of Chemistry, Duke University, Durham, NC 27708 USA

**Keywords:** Engineering, Nanoscience and technology

## Abstract

The addition of surface acoustic wave (SAW) technologies to microfluidics has greatly advanced lab-on-a-chip applications due to their unique and powerful attributes, including high-precision manipulation, versatility, integrability, biocompatibility, contactless nature, and rapid actuation. However, the development of SAW microfluidic devices is limited by complex and time-consuming micro/nanofabrication techniques and access to cleanroom facilities for multistep photolithography and vacuum-based processing. To simplify the fabrication of SAW microfluidic devices with customizable dimensions and functions, we utilized the additive manufacturing technique of aerosol jet printing. We successfully fabricated customized SAW microfluidic devices of varying materials, including silver nanowires, graphene, and poly(3,4-ethylenedioxythiophene) polystyrene sulfonate (PEDOT:PSS). To characterize and compare the acoustic actuation performance of these aerosol jet printed SAW microfluidic devices with their cleanroom-fabricated counterparts, the wave displacements and resonant frequencies of the different fabricated devices were directly measured through scanning laser Doppler vibrometry. Finally, to exhibit the capability of the aerosol jet printed devices for lab-on-a-chip applications, we successfully conducted acoustic streaming and particle concentration experiments. Overall, we demonstrated a novel solution-based, direct-write, single-step, cleanroom-free additive manufacturing technique to rapidly develop SAW microfluidic devices that shows viability for applications in the fields of biology, chemistry, engineering, and medicine.

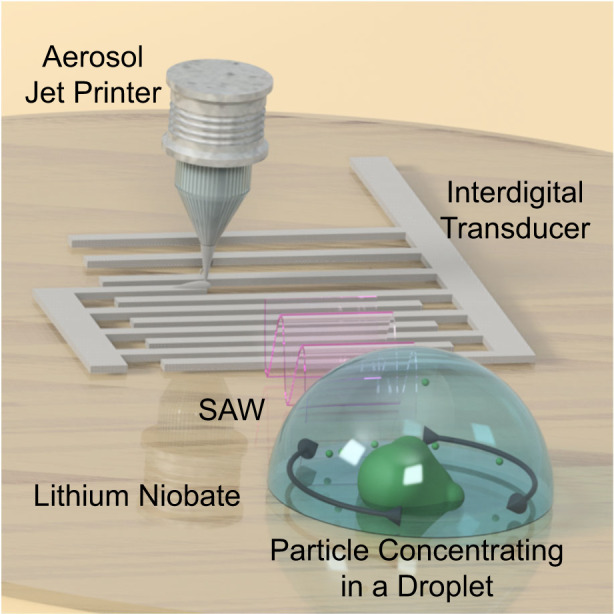

## Introduction

The recent application of surface acoustic wave (SAW) technologies to microfluidics has gained prominence in several fields of research, including biology, medicine, engineering, and materials science^1–6^. SAWs are acoustic waves that travel along the surface of a piezoelectric substrate and are generated by applying a potential to acoustic transducers on the surface of the piezoelectric substrate. The acoustic radiation and streaming forces then propagate into the microfluidic domain, allowing for dynamic manipulation of the fluid and particles/materials within the excited fluid. These SAW-based microfluidic devices offer many powerful features, such as high precision, versatility, a contactless and biocompatible nature, compact devices and accessories, large forces, and fast fluidic actuation. Some particular examples of the breadth of this technology include sample enrichment for sensitive biodetection^7–9^, extracellular vesicle separation and fractionation^8,10–14^, cell‒cell interaction studies^15,16^, cell sorting and separation^17–19^, cellular stimulation^20–22^, patterning^23–27^, droplet manipulation and atomization^28–31^, material manipulation within a droplet^32–37^, intracellular delivery to cells^38–40^, and manipulation of large organisms, including Caenorhabditis elegans and zebrafish larvae^41–43^.

SAW microfluidic devices are typically fabricated through a lengthy, elaborate, and multistep process that requires access to complex and expensive cleanroom equipment^44,45^. The fabrication process often requires several steps, including photolithography, metal evaporation, and chemical lift-off techniques. Photolithography processes add a layer of complexity due to the high-resolution masks that are required to fabricate the microscale interdigital transducer finger widths, which are typically proportional to 1/4^th^ the wavelength of the resonant frequency of the SAW^46–48^. Metal evaporation techniques coupled with the photolithography process also require cleanroom access and are expensive in terms of the cost of use, materials, and high-vacuum equipment itself. Finally, lift-off techniques cannot be utilized for many chemically sensitive piezoelectric substrates. Another cleanroom fabrication technique that can be used to fabricate interdigital transducers that do not require a mask is electron beam lithography. However, electron beam lithography is time-consuming, expensive, may increase the temperature of the substrate, cannot be utilized with chemically sensitive substrates, and also requires access to a cleanroom.

Due to the disadvantages of cleanroom fabrication techniques, several other approaches have been explored for fabricating SAW microfluidic devices, including screen printing^49^, liquid metal molding^50–54^, aluminum foil cutting^55^, and flexible printed circuit board coupling^56–58^. Additive manufacturing methods, such as inkjet printing and aerosol jet printing, provide a maskless and direct fabrication approach for various electrode materials. Recently, aerosol jet printing has been used to fabricate electrodes at a low cost^59^, at high resolutions^60^, does not require any postprocessing fabrication methods^61^, and can print on heated, chemically sensitive, flexible, and/or curved substrates^61,62^. Furthermore, aerosol jet printing has been used to fabricate electrodes using solution-based inks that have been shown to be fully recyclable, carbon-based, biocompatible, and transparent^61,63,64^. Inkjet printing^65–74^ and aerosol jet printing^59,60,75,76^ have been used to fabricate various SAW sensors, but neither method has yet to be proven capable of fabricating working SAW microfluidic devices.

Here, we circumvent conventional cleanroom processes and their accompanying disadvantages by demonstrating the first use of an aerosol jet printer for the rapid fabrication of interdigital transducers for SAW microfluidic applications. We printed planar interdigital transducers on lithium niobate substrates (128° Y-cut) with varying conductive materials, including silver nanowires, graphene, and poly(3,4-ethylenedioxythiophene) polystyrene sulfonate (PEDOT:PSS). These devices were designed to operate at varying frequencies ranging from 5 to 20 MHz. By utilizing aerosol jet printing, we reduced the fabrication time for one interdigital transducer from ~40 h to as little as ~5 min, thereby significantly reducing the device development and customization cycle. The actuated acoustic performance was then measured and directly compared via a vibrometer between the aerosol jet printed SAW microfluidic devices and standard cleanroom fabricated devices. Finally, as a proof-of-concept demonstration, using our printed SAW microfluidic devices, we demonstrated acoustic streaming and particle concentration within a droplet. Using aerosol jet printing, we have demonstrated an additive manufacturing method for SAW microfluidic devices that provides quick, single-step, mask-free, and direct-write fabrication.

## Results

### Aerosol jet printing SAW microfluidic devices

**Fig. 1:**
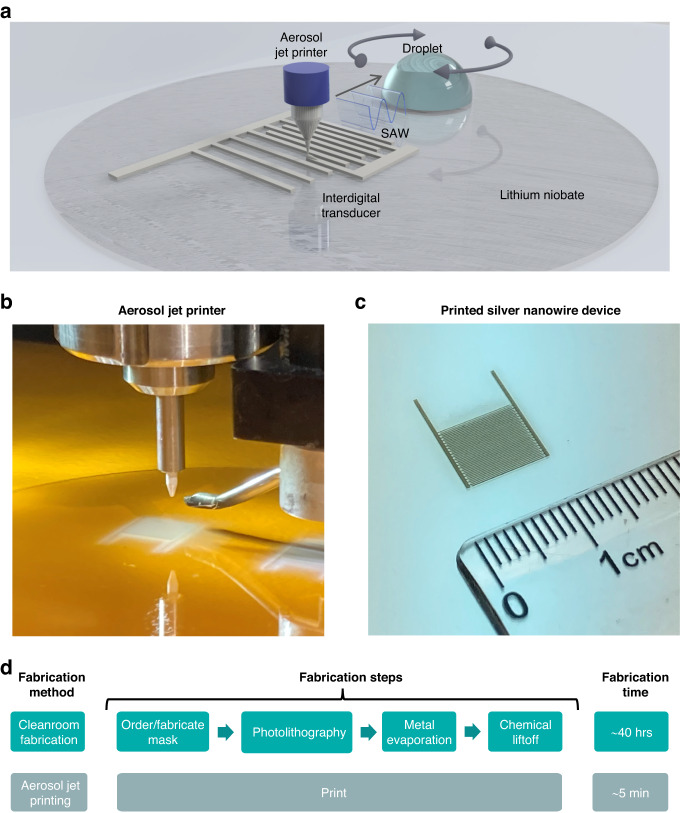
Aerosol jet printing process for surface acoustic wave (SAW) microfluidic devices. **a** Schematic of the fabrication process and mechanism of the aerosol jet-printed SAW microfluidic devices. Interdigital transducers are fabricated via aerosol jet printing and actuated to create SAW that propagates into the droplet to allow for acoustic forces, including acoustic radiation and acoustic streaming, to act on the droplet and the particles inside the droplet. **b** Image of an aerosol jet printer with a printed PEDOT:PSS SAW microfluidic device. **c** Image of a silver nanowire-based interdigital transducer on a lithium niobate substrate. **d** Timeline and number of fabrication steps comparison between the cleanroom and aerosol jet printing fabrication methods for SAW microfluidic devices

As shown by the schematic in Fig. 1a, interdigital transducer patterns are deposited by an aerosol jet printer, and then the resulting devices can immediately actuate SAWs for microfluidic applications, such as acoustic streaming and particle concentration in a droplet. Figure 1b depicts a picture of the Optomec 300 aerosol jet printer, with a printed PEDOT:PSS SAW microfluidic device underneath. To show the microscale resolution of the size of the printed devices, a picture of a silver nanowire device is shown in Fig. 1c with a ruler adjacent to it. Finally, Fig. 1d is a graphic comparison between the time and number of steps required to fabricate a SAW microfluidic device utilizing typical cleanroom fabrication methods versus the aerosol jet printing fabrication method. Assuming it takes 1 day for ordering or fabricating a mask and overnight chemical lift-off after photolithography and vacuum-based metal evaporation; with our method, both the amount of time and the number of steps are greatly reduced, from ~40 h to as little as 5 min and 4 steps to 1 step. These features exhibit how aerosol jet printing can effectively be used to rapidly create customizable SAW microfluidic devices.

**Fig. 2:**
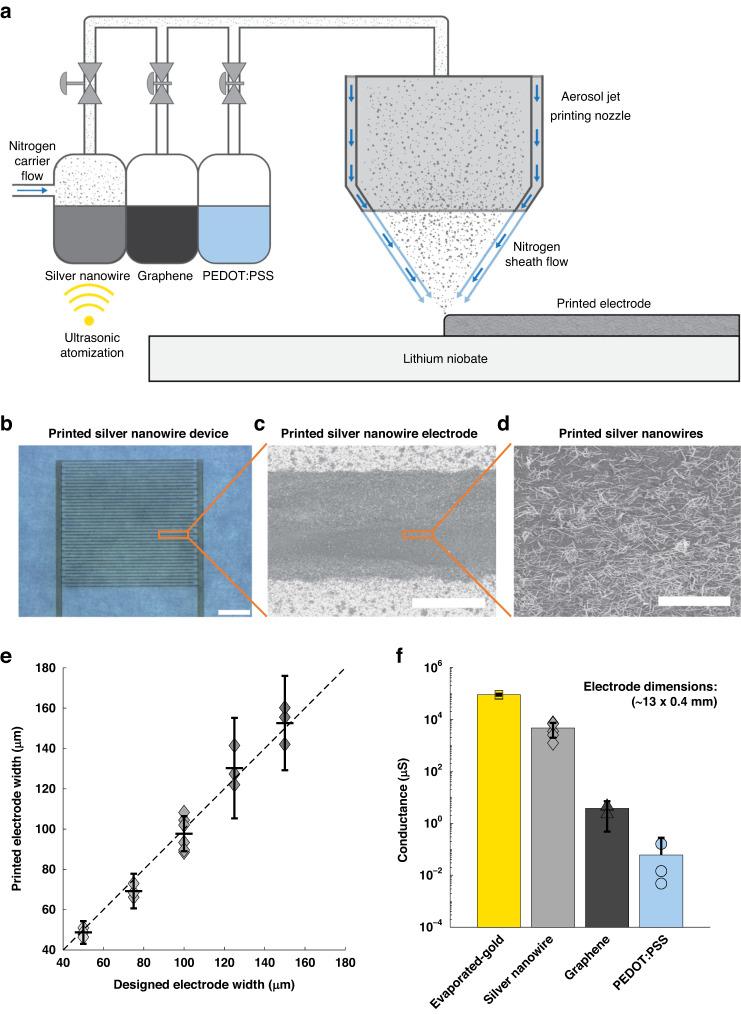
Aerosol jet printing interdigital transducers (IDTs) with different electrode widths and materials for SAW microfluidics. **a** Schematic of the aerosol jet printing process for manufacturing IDTs. The selected electrode material suspension (i.e., ink) is aerosolized via ultrasonication and concentrated by nitrogen sheath flow to print from the nozzle. **b** Picture of an aerosol jet-printed silver nanowire SAW microfluidic device. The scale bar is ~2 mm. **c**, **d** Scanning electron microscope images of printed silver nanowire electrodes at increasing magnification. The scale bar for (**c**) is 50 μm, and that for (**d**) is 5 μm. **e** Comparison of actual (data points) and ideal (dotted line) versus designed printed electrode widths averaged across devices. Sample sizes are 3 different devices, except for the 100 μm devices that have 6. Error bars represent the 95% confidence interval for the designed electrode width, and the—symbol represents the mean printed electrode width. **f** Comparison of two-terminal conductance to material type for fabricated SAW microfluidic devices, including evaporated-gold and aerosol jet printed materials, including silver nanowire, graphene, and PEDOT:PSS. The electrode dimensions are ~13 x 0.4 mm (LxW), and the respective sample sizes are as follows: evaporated gold = 4, silver nanowire = 6, graphene = 3, and PEDOT:PSS = 3. Error bars represent the 95% confidence interval. Note that PEDOT:PSS does not have a bottom error bar because a negative error bar cannot be displayed in a log-axis plot

The aerosol jet printing fabrication process is depicted in the schematic of Fig. 2a. The conductive ink is atomized via ultrasound atomization and transported to the nozzle by a nitrogen carrier gas flow and is then focused and deposited by a secondary annular nitrogen sheath flow in the 150 μm aerosol printing nozzle. An example of a printed silver nanowire SAW microfluidic device is shown in Fig. 2b, and all other devices are shown in Supplemental Fig. 1 and Supplemental Fig. 2. Examination of a single electrode in the silver nanowire device at high magnification, as shown in Fig. 2c, reveals the silver nanowires making up the electrodes. At higher magnification (Fig. 2d), the fine details of the silver nanowires within the electrode become apparent. The average electrode widths for each aerosol-printed SAW microfluidic device were measured optically and are depicted in Fig. 2e. As shown by the 95% confidence intervals of the average printed electrode width of multiple devices for the different designs, the aerosol jet printer can deposit electrode finger widths over the entire desired range with higher accuracy in the smaller sized devices and moderate accuracy for the larger devices. The reduction in electrode-width accuracy may be attributed to the additional build-up of overspray, a phenomenon where errant ink particles are deposited away from the majority of the ink deposition^77^. The different measured widths for the different materials are shown in Supplemental Fig. 3a and show similar variability.

To characterize and compare the electrical performance of the SAW microfluidic devices printed with different materials, electrode conductivity was measured. As shown in Fig. 2f, the silver nanowire-based SAW microfluidic devices offered the greatest conductivity among the printed materials, which is attributed to the overlapping of the metallic materials throughout the printed interconnected network of the electrode, as shown in Fig. 2c. However, compared with the cleanroom-fabricated SAW microfluidic devices, the evaporated-gold devices offered 45x greater conductivity than the nonsintered, loosely connected, silver nanowire-printed devices. If a higher conductivity silver nanowire device was desired, the silver nanowire electrodes could undergo an additional sintering process^78–80^. The conductivity measurements of the different printed silver nanowire width devices are shown in Supplemental Fig. 3b and show similar conductivities. Although the graphene and PEDOT:PSS devices had lower conductivities than the silver nanowire printed devices, with further device optimization, the thin and light graphene can mitigate mass-loading effects to reduce the inertia of the interdigital transducer^81–83^, and PEDOT:PSS could be further utilized for transparent interdigital transducers, as shown by the images in Supplemental Fig. 1c.

### Acoustic characterization and comparison

**Fig. 3:**
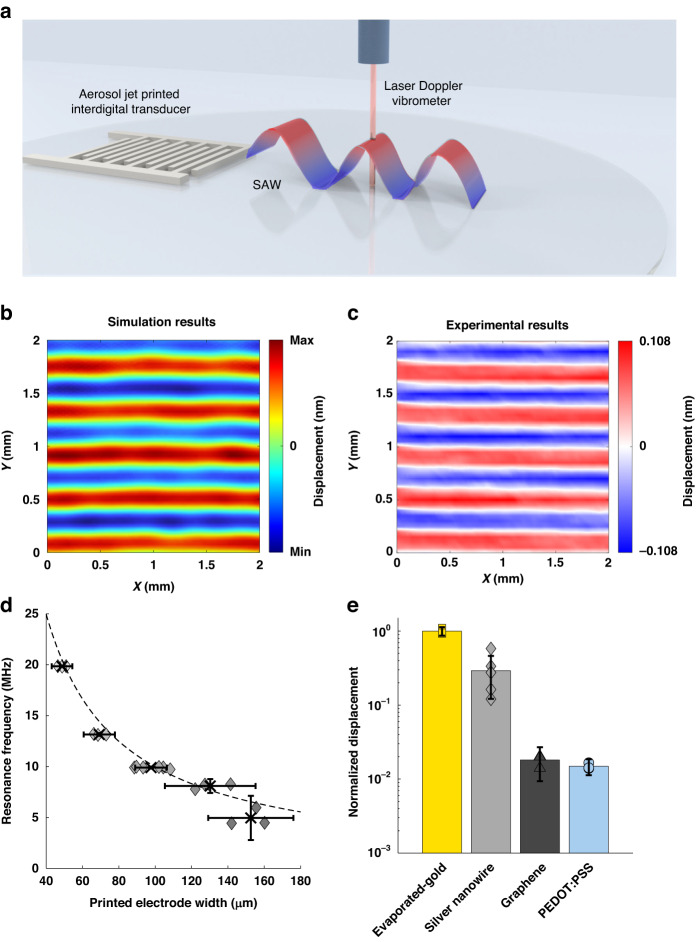
Acoustic response and comparison across the different materials and configurations of printed SAW microfluidic devices. **a** Schematic of acoustic characterization of a printed SAW microfluidic device, where a vibrometer is focused on the top surface of a lithium niobate substrate to measure the local displacement of a propagating surface acoustic wave based on the Doppler effect. Moving the laser focusing spot to different positions yields a displacement field of waves. **b** Numerically simulated displacement field of a traveling surface acoustic wave generated by a planar interdigital transducer at 9.98 MHz. **c** Experimentally measured displacement field in a 2 x 2 mm area in front of a silver nanowire printed interdigital transducer with a designed interdigital finger width of 100 μm. The max and min values on the scale bar are the averaged max and min displacements of that device. **d** Comparison of measured resonant frequencies (data points) and theoretical resonant frequency (dotted line) versus printed interdigital electrode widths. Sample sizes are 3 different devices, except for the 100 μm silver nanowire devices with 6. Error bars represent the 95% confidence interval, and the x symbol represents the mean resonant frequency and printed electrode width. **e** Displacement of the different SAW microfluidic devices, normalized by the ratio of the minimum electrical power output (32.8 Vpp) and the electrical power output of the measured device. Displacement is also normalized to the mean maximum displacement of the evaporated-gold devices (0.65 nm). Sample sizes: evaporated gold = 4, silver nanowire = 6, graphene = 3, and PEDOT:PSS = 3. Error bars represent the 95% confidence interval

To characterize and compare the acoustic performance between the printed SAW microfluidic devices and the typical cleanroom fabricated devices, we utilized a laser Doppler vibrometer that can measure periodic surface displacements at the laser spot based on the Doppler effect. Using this laser vibrometry setup as shown in Fig. 3a, we measured each SAW microfluidic device’s surface displacements at multiple wave generation frequencies to determine the resonant frequency with the maximum displacement. By moving the laser spot to different positions to perform point-by-point measurements, we obtained a 2D displacement field to visually show the generated SAWs. The measured acoustic displacement field for a silver nanowire device is shown in Fig. 3c, in which the measured displacement field pattern is similar to the simulated acoustic displacement field pattern (Fig. 3b) of the corresponding interdigital transducer design. As shown in Fig. 3d, the different measured resonant frequencies for the different printed electrode widths match up very closely to the theoretical resonant frequencies (dotted line) for the printed silver nanowire devices, thus showing the reliability of aerosol jet printing for fabricating SAW microfluidic devices of varying frequencies. This effect is also observed in Supplemental Fig. 5, which shows the network analyzer S11 response of the different frequency silver nanowire SAW microfluidic devices and that the measured resonant frequency from the network analyzer tends to match both the measured resonant frequency from the laser Doppler vibrometer and the theoretical resonant frequency. This outcome is attributed to the accurate periodicity of both the printed electrode width and gap width, which is defined by the stepping accuracy of the aerosol jet printing system. Because the resolution of the aerosol jet printer is 10 μm, the theoretical frequency limit of aerosol jet-printed SAW microfluidic devices is ~99.8 MHz on 128° Y-cut lithium niobate. However, achieving this theoretical frequency limit with reliable device yields will require further optimization.

The measured resonant frequencies of the different material SAW microfluidic devices are shown in Supplemental Fig. 4a. The measured resonant frequencies of the graphene and PEDOT:PSS devices (6.95 and 7.03 MHz, respectively) are less than anticipated (9.98 MHz), which may be attributed to the distortion of the waveform with the high-power input into the amplifier that is required to attain a signal. Finally, the comparison between the normalized displacement of the different material printed devices and cleanroom fabricated devices is shown in Fig. 3e. The normalized displacements for different printed configurations are shown in Supplemental Fig. 4b. As anticipated, the measured displacement decreases with the decrease in conductance due to the increase in resistive power loss^84^. This can also be observed in the different output magnitudes measured from the network analyzer S11 response of the different material SAW microfluidic devices, as shown in Supplemental Fig. 6. Other factors, such as mass loading, frequency, capacitance, and inductance, can also influence the measured displacement^48,81,85,86^. Although the cleanroom fabricated device shows the best performance, the performance of the silver nanowire printed device is still favorable. Furthermore, although the printed graphene and PEDOT:PSS devices had a lower conductivity and displacements than the silver nanowires, the acoustic displacement fields for the graphene and PEDOT:PSS-based devices remained detectable, thereby showing their utility for SAW microfluidic applications.

### Demonstration of SAW microfluidic functions of the aerosol jet printed devices

**Fig. 4:**
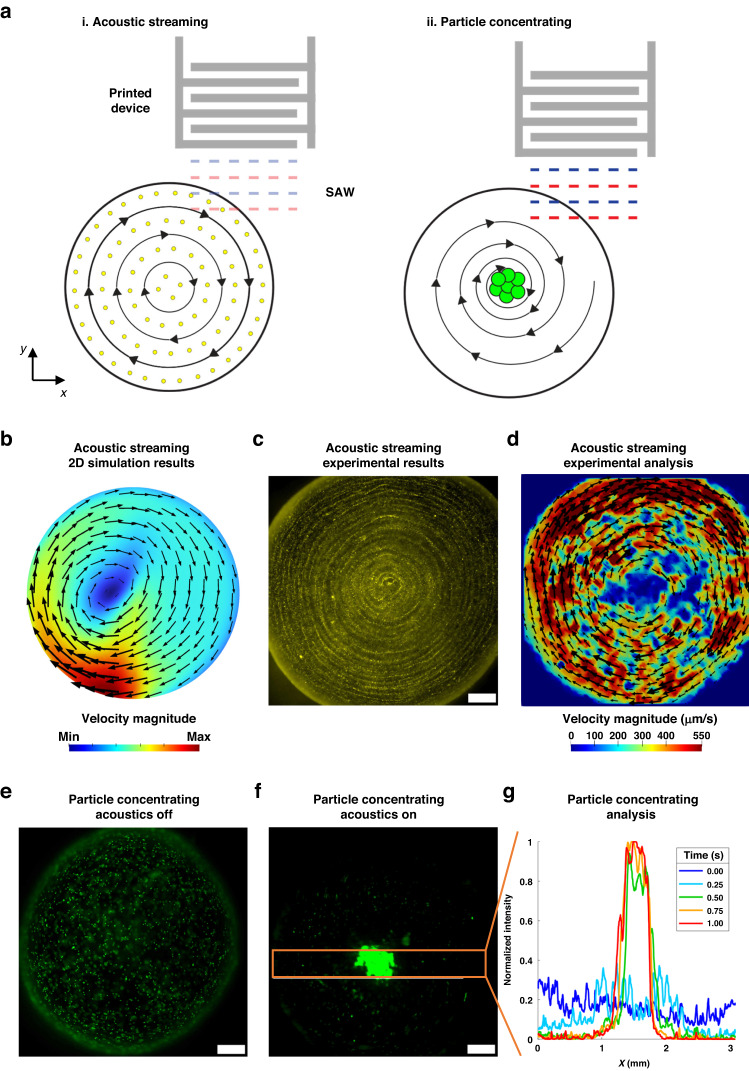
Microfluidic demonstrations of various printed SAW microfluidic devices. **a** Schematics of the two SAW microfluidic demonstrations, including (i) acoustic streaming and (ii). Particle concentrating, with their respective acoustic wave amplitudes depicted via opacity. **b** Numerically simulated fluid streaming velocity magnitude and distribution within a droplet with an applied body force (SAWs) applied to one half of the droplet. **c** Image of 2 μm particle streaming in a 5 μL droplet via a silver nanowire device, with an output power of 65.2 Vpp. **d** Particle image velocimetry analysis of (**c**). **e**–**g** 10 μm particle concentration experiment in a 5 μL droplet using a silver nanowire device, with an output power of 88.8 Vpp, with (**e**) acoustics off and (**f**) acoustics on after 1 s of acoustic power input. **g** Normalized fluorescence intensity of the highlighted region of interest, as depicted by the orange box in (**f**), over a period of 1 s after the acoustics were initially actuated. All scale bars are 400 μm

To exhibit the aerosol jet printed devices for different SAW microfluidic applications, we demonstrated acoustic streaming and particle concentration, as shown in Fig. 4. Figure 4a is a 2D schematic of the two different demonstrations. As the offset droplet is excited by the incident traveling SAW, a combination of acoustic radiation forces and acoustic streaming forces act on the droplet. Depending on the power of the SAW, as depicted by the change in opacity of the SAW between Fig. 4a i–ii, and the size of the particle, different microfluidic applications can be achieved. The fluid velocity magnitude and distribution within the droplet in the aerosol jet printed SAW microfluidic devices is depicted in Fig. 4b. As shown in the simulation of Fig. 4b, the acoustic vortex center is slightly offset to the center of the droplet. This most likely occurs due to the asymmetric force generated by the traveling SAW that incidents on one half of the droplet. The 3D streaming and particle tracing simulations within a droplet are shown in Supplemental Fig 7. The acoustic particle streaming phenomenon is observed experimentally in the microscope image in Fig. 4c and the respective Supplemental Movie 1 by using a 100 μm finger width silver nanowire aerosol jet printed device. To achieve acoustic streaming, an output voltage of 65.2 Vpp was needed. Particle image velocimetry was then applied to measure the acoustic streaming velocity field within the droplet, averaging ~550 μm/s, as seen in Fig. 4d. The measured acoustic streaming velocity distribution of Fig. 4d is comparable to the simulated fluid velocity distribution in Fig. 4b and demonstrates the capability of aerosol jet-printed SAW microfluidic devices.

To further exhibit the SAW microfluidic functionality of the aerosol jet printed devices, we detected the concentration of 10 μm particles within a droplet over time utilizing a 100 μm finger width silver nanowire device, as shown in Fig. 4e–g and Supplemental Movie 2. Figure 4e shows the particle distribution within the droplet with the acoustics off, and Fig. 4f shows the particle distribution within the same droplet 1 s after the acoustics were actuated. To achieve particle concentration, an output voltage of 88.8 Vpp was required due to the higher power that is required to achieve this phenomenon. The mechanism behind particle concentration within a droplet has been explored previously^32–37^ and relies on a combination of acoustic streaming force, acoustic radiation force, and centrifugal forces that are generated from the traveling surface acoustic waves and standing surface acoustic waves within the droplet. These factors are dependent on the acoustic attenuation length, the frequency of the SAWs, the size of the particle, the density of the particle, the acoustic power applied, the size of the droplet, and the contact angle of the droplet. The recording of the concentration versus time is shown in Fig. 4g. Over time, the normalized fluorescence intensity increases in the center of the droplet, showing how the particles concentrate within the droplet. The combination of these SAW microfluidic demonstrations shows how aerosol jet printing can be used to fabricate effective and functional SAW microfluidic devices.

## Discussion

In this work, we developed SAW microfluidic devices fabricated via aerosol jet printing. To demonstrate the versatility of the aerosol jet printer for SAW device fabrication, we printed electrodes comprised of varying materials, including silver nanowires, graphene, and PEDOT:PSS, and designed the resulting electrodes for a range of different acoustic frequencies. We then analyzed and measured the acoustic displacement and resonant frequencies of the aerosol jet-printed SAW microfluidic devices via a laser Doppler vibrometer and compared the acoustic performance of the printed devices to that of cleanroom-fabricated devices. Finally, to exhibit SAW microfluidic functionality with the printed devices, we performed particle streaming and concentrating measurements. Using aerosol jet printing, we have demonstrated an additive manufacturing technique that provides a direct, single-step, and maskless fabrication method for enabling rapid fabrication of customizable SAW microfluidic devices. Potential future works include printing devices on heat-/chemical-sensitive piezoelectric substrates, printing devices with various acoustic transducer designs, printing higher frequency devices, printing bulk acoustic wave microfluidic devices, and printing on curved surfaces for various applications in biology, medicine, engineering, and materials science.

## Materials and methods

### Aerosol jet printing SAW microfluidic devices

All printed materials were biocompatible, recyclable, and water-based conductive inks. The silver nanowire ink was formulated by adding 100 µL of 1 w/w% 66 kDa hydroxypropyl methylcellulose (HPMC) polymer (9004-65-3, Sigma Aldrich, USA) in 18.2 MΩ deionized water to an aqueous suspension containing 900 μL of 5 mg/mL 20 nm x 12 μm silver nanowires (806609, Sigma Aldrich, USA). This is due to a significant increase in electrode quality, as described in prior work^61^. To develop graphene interdigital transducers, a 10 w/w% graphene ink (808261-10ML, Sigma‒Aldrich, USA) was diluted to ~2.3 w/w% with distilled water before printing. The poly(3,4-ethylenedioxylthiophene)-poly(styrene-sulfonate) (PEDOT:PSS) ink was produced by adding 100 μL of 1 w/w% 66 kDa HPMC to 0.9 mL of an aqueous 3.0–4.0 w/w% high conductivity grade PEDOT:PSS (655201, Sigma Aldrich, USA) solution.

All conductive inks were deposited by means of an aerosol jet printer (AJ300, Optomec, USA) with a 150 μm inner-diameter nozzle (8002171, Optomec, USA). A sheath flow of 30–32 standard cubic centimeters (SCCM) was used for all materials. To maintain a single-line width of ~34 μm, the atomizer flow rate varied from 32–36 SCCM for all materials. The atomizer was held at room temperature for all atomized suspensions, except that the ink bath temperature was set at 50 °C for printing all graphene interdigital transducers. All conductive ink microdroplets were atomized using a 370–380 mA ultrasonic inducer current, and all devices were deposited onto the surface of a lithium niobate wafer with the platen temperature maintained at 80 °C to ensure a fast ink-drying time. All materials were printed with 1 pass at a print speed of 4 mm/s. The devices were ready to use immediately without any further postprocessing needed.

The cleanroom fabricated SAW microfluidic devices for performance comparison were fabricated utilizing standard positive photolithography patterning, e-beam evaporation, and lift-off methods. First, photoresist (SPR3012, Kayaku, Japan) was spin coated onto a lithium niobate substrate (PWLN-431232, Precision Micro-optics, USA) at 5000 rpm and then baked at 95 °C for 90 s on a hot plate. After being exposed to 365 nm UV light with 12 mJ/cm^2^ for 7 s (MA6/BA6, Suss Microtech, Germany) with the aligned mask (artnet pro Inc., USA), the sample was baked for 1 min at 115 °C for 90 s. Then, the sample was developed for 60 s in MF CD-26 developer (Kayaku, Japan). Following the lithography steps, 20 nm of chrome and 80 nm of gold were evaporated sequentially by e-beam evaporation (CHA Industries Solution E-Beam, USA) with evaporation rates of 5 Å/s and 2 Å/s, respectively. The liftoff process was achieved by placing the completed wafer in acetone for at least overnight and removing the excess gold and photoresist.

The electrode design for the SAW microfluidic device was designed with that of a planar interdigital transducer design. Each design had 20 interdigital finger pairs, equal finger and gap spacing, and 2 finger width distances between the finger edge and the edge of the bars, and the length of the interdigital transducers was approximately the width of the SAW microfluidic device with 20 interdigital finger pairs. To connect the fabricated SAW microfluidic devices to the electrical equipment, wires were adhered to the printed interdigital transducer bars via silver epoxy.

### Electrical and optical characterization of the aerosol jet-printed SAW microfluidic devices

To determine the conductance of the different electrodes of the printed devices, conductivity was measured by means of a 2-point probe method, through which precision micromanipulators and a signal measurement unit (B2902A, Keysight, USA) were employed. The current versus time was monitored as a stepwise voltage input of 1 mV, 10 mV, and 100 mV was applied, and each potential was held constant for 3 s, resulting in a total measurement time of 9 s. Three experiments were performed across the length of each interdigital transducer bar with a width of ~0.4 mm, and the probes were positioned at slightly different locations before taking each measurement. The slight conductance variations are likely due to variations in the contact resistance between the probe and the deposited film. Broad field-of-view images of the devices were captured using a low-magnification optical microscope (SZ61, Olympus, USA) equipped with a camera (Axiocam 105 Color, Zeiss, USA), and the printed electrode width was measured with 2.5x and 10x lenses using a calibrated optical microscope (AX10, Olympus, USA). The average printed line width for each device was measured by averaging the top 5 and bottom 5 electrode widths. High-resolution images of the 20 nm x 12 µm silver nanowire materials on the lithium niobate substrate were obtained by scanning electron microscopy (Apreo S, ThermoFisher Scientific, USA) at the Shared Materials Instrumentation Facility at Duke University.

### Acoustic simulations

The simulation of the piezoelectric phenomenon was conducted in COMSOL MULTIPHYSICS 5.6 by fully coupling the electrostatics module and solid mechanics module through the piezoelectric effect interface. The material properties of the Y128 lithium niobate were obtained from published works^87^. To simulate the surface acoustic wave propagation on an anisotropic Y 128° lithium niobate substrate, 20 periodic printed pairs of fingers (8.2 mm × 100 μm, 100 μm gap between fingers) were modeled on a 0.5 mm thick lithium niobate substrate. A voltage port V0 was applied on half of the positive electrodes, and a 0 V ground port was applied on the ground electrodes. The surrounding edges of the substrate were set as low reflection boundaries. The model was run at 9.98 MHz in a frequency domain solver, and the out-of-plane displacement was plotted for comparison with the experimental result.

The simulation of the fluid streaming velocity within a droplet was conducted in COMSOL MULTIPHYSICS 5.6 with a fluid mechanical module. To simulate the acoustic streaming velocity distribution and magnitude within a droplet, a body force^88,89^ was applied to one half of a 3D water droplet with a diameter of 4 mm and a height of 1.06 mm. The body force can be expressed as:

$${F}_{z}=-\rho \left(1+{\alpha }^{2}\right){A}^{2}{\omega }^{2}{k}_{i}\alpha {e}^{2({k}_{i}x+2{k}_{i}z)},$$

$${F}_{x}=-\rho \left(1+{\alpha }^{2}\right){A}^{2}{\omega }^{2}{k}_{i}{e}^{2({k}_{i}x+2{k}_{i}z)}.$$

where *ρ* is the water density, *α* = 2.47 is the attenuation coefficient, *A* = 1 nm is the amplitude, *ω* is the angular frequency, *k*_i_ = *ω*/*c*_*l*_ is the imaginary part of the wavenumber, and *c*_*l*_ = 3931+i*68.1 m/s is the wave speed of the leaky surface acoustic wave in water^43,88^. The fluid pressure at a random point at the bottom of the droplet is set to 0 Pa to help the computation converge faster. The model was calculated with a stationary solver. The bottom of the droplet was set as a no-slip condition, and the open edges of the droplet were set as slip conditions. A cross-Section 100 μm above the bottom of the 3D acoustic streaming simulation in Supplemental Fig. 7a is shown in Fig. 4b.

The simulation of 3D particle tracing, as shown in Supplemental Fig. 7b, was conducted in COMSOL MULTIPHYSICS 5.6 with the particle tracing module, in which the Stokes drag force and gravity force were applied on particles (diameter of 2 μm, density of 1180 kg/m^3^). Forty particles were placed in random positions within the droplet domain at time *t* = 0 s and calculated at a time step of 0.01 s. The particle tracing figure was plotted at *t* = 0.25 s.

### Vibrometer measurements and analysis

A laser Doppler vibrometer (VFX-1–130, Polytec, USA) was used to acquire the displacements of the generated acoustic waves by focusing the laser spot on the top surface of the piezoelectric substrate. For each SAW microfluidic device, point-by-point measurements were performed at multiple positions on a 2D grid (with a spatial resolution of 0.04 mm) in a 2 x 2 mm area in front of the transducer. The acoustic signal was generated by a function generator (DG1022, Rigol, China) and amplified (A150, E&I Pure Power, USA) to enhance the signal, and the corresponding acoustic signal voltage was measured by an oscilloscope (SDS1202X-E, Siglent, China) and converted to the displacement according to the Doppler vibrometer sensitivity, which was set to 10 nm/V for all experiments. The vibrometer path parameters for the amplitude and resonant frequency measurements were designed to be 3 wavelengths long in the y-direction (y-distance ranging from 0.6 to 1.8 mm), measured at least 3 times in the x-direction (x-distance set to 0.05 mm with a spatial resolution ranging from 0.02–0.03 mm), with at least 10 measurements per wavelength. Later, a 1.25 scaling offset was measured for the *x*–*y* stage movement and was applied to the x- and y-coordinate information in Fig. 3c.

The SAW microfluidic devices were actuated by a 3-cycle Hanning window modulated sine signal with a center frequency near each device’s design frequency (i.e., 19.92, 12.6, 10.5, 7.8, and 4.2 MHz were applied for the silver nanowire printed devices with electrode widths of 50, 75, 100, 125, and 150 μm, respectively). The excitation center frequency applied for the 100 μm wide cleanroom fabricated SAW microfluidic device and the 100 μm printed silver nanowire device was 10.5 MHz. The excitation center frequency applied for the 100 μm wide graphene and PEDOT:PSS printed devices was 7.5 MHz, as their resonant frequency was measured to be lower than the expected resonant frequency, as shown in Supplemental Fig. 4a. The output voltage of the function generator applied for the silver nanowire and cleanroom fabricated devices was 40 mVpp, except for the 150 μm silver nanowire printed device, which was 200 mVpp, and the output voltage of the function generator applied for the graphene and PEDOT:PSS devices was 1.5 Vpp. Each device was amplified to acquire wave signals with good signal-to-noise ratios. Later, the electrical output of the amplifier was measured to be 32.8 Vpp, 128 Vpp, and 424 Vpp for the aforementioned function generator outputs for an input frequency of 10.5 MHz.

After the acoustic signal was attained, the signal was then filtered to minimize noise. A filter was first applied to center the signal to account for any noise offset in the acquired signal. This was done by subtracting the original signal from the average value of that signal. A frequency filter was then applied to reduce any noise that originated from frequencies other than the applied resonant frequency. This was done by transferring the acquired time-domain signal to the frequency domain by a fast Fourier transform, finding the maximum resonant frequency peak, setting the signal to zero that was outside of a bandwidth that was 40% of the measured resonant frequency in width and was centered at the resonance frequency, and then applying an inverse Fourier transform to obtain the frequency filtered time-domain signal. The average maximum absolute displacement of each filtered signal was then calculated by averaging the peak heights that were at least 80% of the height of the maximum peak of the signal. The average maximum absolute displacement of each signal was then averaged to acquire the average maximum absolute displacement for each SAW microfluidic device. Each SAW microfluidic average maximum absolute displacement was then normalized by the ratio of the minimum electrical power output (32.8 Vpp) and the electrical power output of the measured device. The displacement was also normalized to the mean maximum displacement of the evaporated-gold devices (0.65 nm). The average resonant frequency of each SAW microfluidic device was calculated by averaging the resonant frequency of each signal.

### SAW microfluidic experiments and analysis

The optical setup for the SAW microfluidic experiments was conducted using an upright microscope (BX51WI, Olympus, Japan), and images and videos were acquired using an attached CMOS camera (Zyla 4.2 sCMOS, Andor, USA). The electrical setup for the SAW microfluidic experiments included a function generator (E4411B, Agilent, USA), amplifier (25A250A, Amplifier Research, USA), impedance matcher (MFJ-974HB, MFJ Enterprises Inc., USA), and an oscilloscope (2190E, BK Precision, USA). The function generator, amplifier, and impedance matcher were connected in series via 50 Ω coaxial cables, which were then connected to the wires of the printed SAW microfluidic devices with a 50 Ω coaxial cable and a pair of clips. Also connected to the wires of the SAW microfluidic device was a 100x oscilloscope probe, which was connected to the oscilloscope. The impedance matcher parameters (i.e., antenna, inductance, and transmitter) were held at a constant for each device at 1, B, and 1 for the respective parameters.

The particles used for particle patterning and acoustic streaming were 2 μm yellow‒green, fluorescent polystyrene particles (PSYF002UM, Magsphere, USA), and the particles used for concentrating within the droplet were 10 μm yellow‒green, fluorescent polystyrene particles (PSYF010UM, Magsphere, USA). The particles were suspended in distilled water (UltraPure Distilled Water, Life Technologies, USA), and a 5 μL droplet was utilized for each experiment. The droplets were placed in front of the left corner of the SAW microfluidic device in each experiment. The SAW microfluidic concentration quantification of fluorescence intensity was measured every 4 frames per second, or 250 ms, starting with time 0 as the first observed movement of particles with the acoustics on. The region of interest shown in the microsphere image in Fig. 4h was the same region of interest in which the signal intensity was analyzed in ImageJ and plotted as a profile over distance for each time in Fig. 4g.

### Particle image velocimetry analysis

To measure the acoustic streaming flow field in the droplet, a particle image velocimetry (PIV) method was used. Particle image velocimetry images of the 2 μm yellow‒green, fluorescent tracer polystyrene particles (PSYF002UM, Magsphere, USA) were captured with a CMOS camera (Zyla 4.2 sCMOS, Andor, USA) at a frame rate of 20 fps via a 4x objective lens. The average flow field and velocity vectors were acquired using a PIV software package (Flow Expert, KATO Koken, Japan) that measured the change in the particle location between the sequential frames using an interrogation window of 48 x 48 pixels (78 x 78 μm).

### Supplementary information

Revised Supplemental Material

Supplemental Movie 1

Supplemental Movie 2
