# Supplementary material for: Aerosol jet printing of surface acoustic wave microfluidic devices
Source: Microsyst Nanoeng. 2024 Jan 1;10:2. doi: 10.1038/s41378-023-00606-z (PMC10757899; doi:10.1038/s41378-023-00606-z)
Supplement: Supplementary file 1 — Revised Supplemental Material [file 41378_2023_606_MOESM1_ESM.docx]

**Aerosol Jet Printing Surface Acoustic Wave** Microfluidic Devices

Joseph Rich^1#^, Brian Cole^2#^, Teng Li^3^, Brandon Lu^1^, Hanyu Fu^1^, Brittany Smith^2^, Jianping Xia^4^, Shujie Yang^4^, Ruoyu Zhong^4^, James L. Doherty^2^, Kanji Kaneko^5^, Hiroaki Suzuki^5^, Zhenhua Tian^3*^, Aaron Franklin^2,6*^, Tony Jun Huang^4*^

^1^Department of Biomedical Engineering Duke University, Durham, NC, 27708, USA

^2^Department of Electrical and Computer Engineering Duke University, Durham, NC, 27708, USA

^3^Department of Mechanical Engineering, Virginia Polytechnic Institute and State University, Blacksburg, VA 24061, USA

^4^Thomas Lord Department of Mechanical Engineering and Materials Science, Duke University, Durham, NC 27708, USA

^5^Deptartment of Precision Mechanics, Faculty of Science and Engineering, Chuo University, Tokyo 112-8551, Japan

^6^Department of Chemistry, Duke University, Durham, NC 27708, USA

^#^These authors contributed equally to this work.

*Corresponding authors: Zhenhua Tian ([tianz@vt.edu](mailto:tianz@vt.edu)), Aaron D. Franklin ([aaron.franklin@duke.edu](mailto:aaron.franklin@duke.edu)), and Tony Jun Huang ([tony.huang@duke.edu](mailto:tony.huang@duke.edu))

**Keywords:** Acoustics, Microfluidics, Aerosol jet printing, Surface acoustic waves, Additive manufacturing


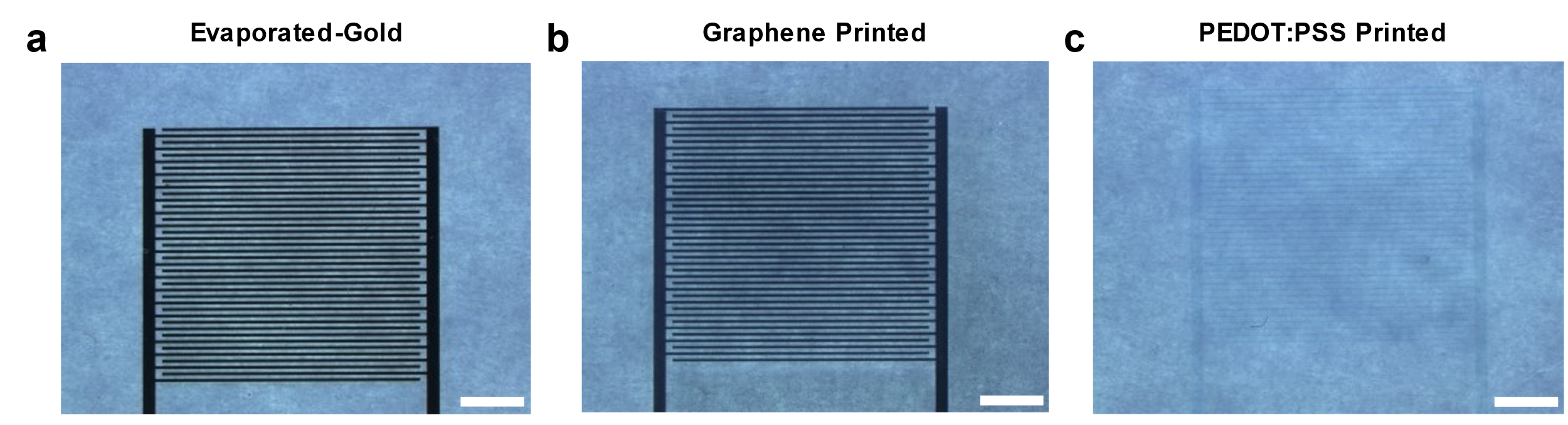


**Supplemental Figure 1.** Images of the 100 μm electrode width surface acoustic wave (SAW) microfluidic devices of different materials. Pictures of (a) evaporated-gold fabricated device, (b) graphene aerosol jet printed device, and (c) PEDOT:PSS aerosol jet printed device. All scale bars are ~2 mm.


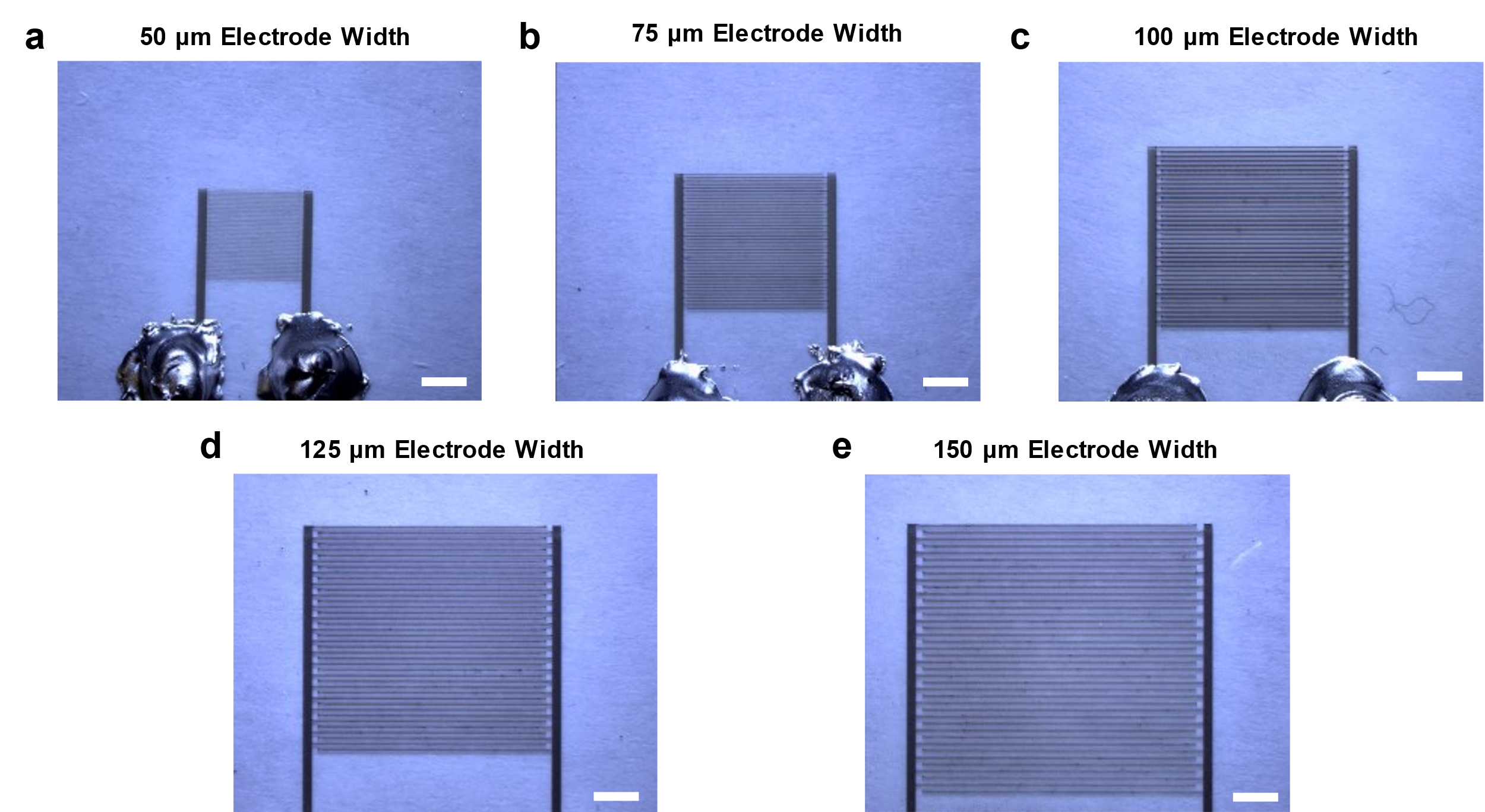


**Supplemental Figure 2.** Images of printed silver nanowire SAW microfluidic devices with different electrode widths of (a) 50 μm, (b) 75 μm, (c) 100 μm, (d) 125 μm, and (e) 150 μm. All scale bars are ~2 mm.


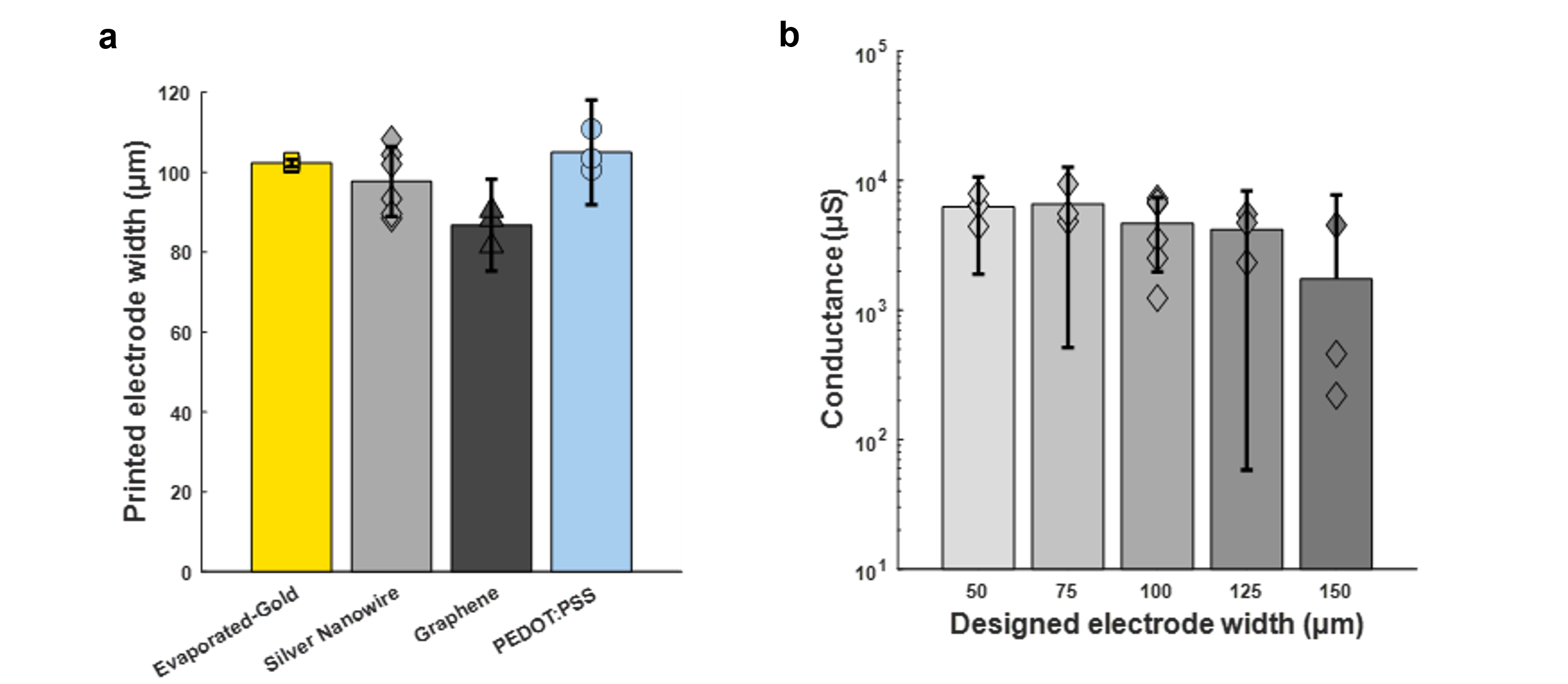


**Supplemental Figure 3.** Optical and electrical characteristics of different SAW microfluidic devices. (a) **Optically measured interdigital electrode width for different materials, with the plotted bars represented averages. All electrodes were designed to be 100 µms in width. Sample sizes are as follows: evaporated-gold = 4, silver nanowire = 6, graphene = 3, and PEDOT:PSS = 3. Error bars represent 95% confidence interval.** (b) **Two-terminal conductance measurements of the different printed interdigital electrode width devices. Measured electrode dimensions are ~9, 11, 13, 15, 17 x 0.4 mm (LxW) for the respective 50, 75, 100, 125, and 150 μm designed electrode width devices. Sample sizes are 3 different devices, except for the 100 μm silver nanowire devices that have 6. Error bars represent the 95% confidence interval. Note that 150 μm device does not have a bottom error bar, because a negative error bar cannot be displayed in a log-axis plot.**


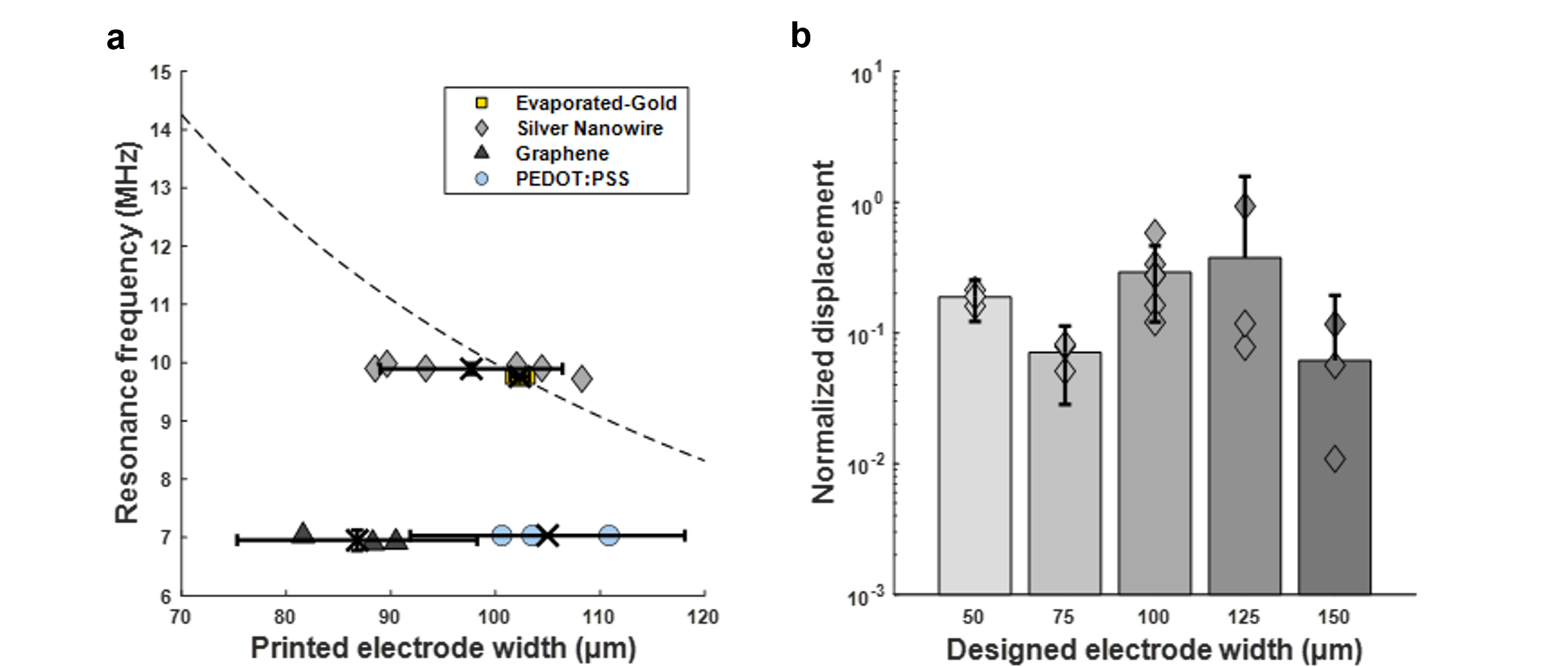


**Supplemental Figure 4.** **Acoustic response and comparison across the different materials and configurations of the printed SAW microfluidic devices**. **(a) Comparison of measured resonant frequencies (data points) and the theoretical resonant frequency (dotted line) of the different printed interdigital electrode materials. Sample sizes are as follows: evaporated-gold = 4, silver nanowire = 6, graphene = 3, and PEDOT:PSS = 3. Error bars represent the 95% confidence interval, and the x symbol represents the mean resonant frequency and printed electrode width. (b) Displacement of the different SAW microfluidic devices, normalized by the ratio of the minimum electrical power output (32.8 Vpp) and the electrical power output of the measured device. Displacement is also normalized to the mean maximum displacement of the evaporated-gold devices (0.65 nm) in Figure 3e. Sample sizes are 3 different devices, except for the 100 μm silver nanowire devices that have 6. The error bars represent the 95% confidence interval. Note that 125 μm and 150 μm device do not have a bottom error bar, because a negative error bar cannot be displayed in a log-axis plot.**


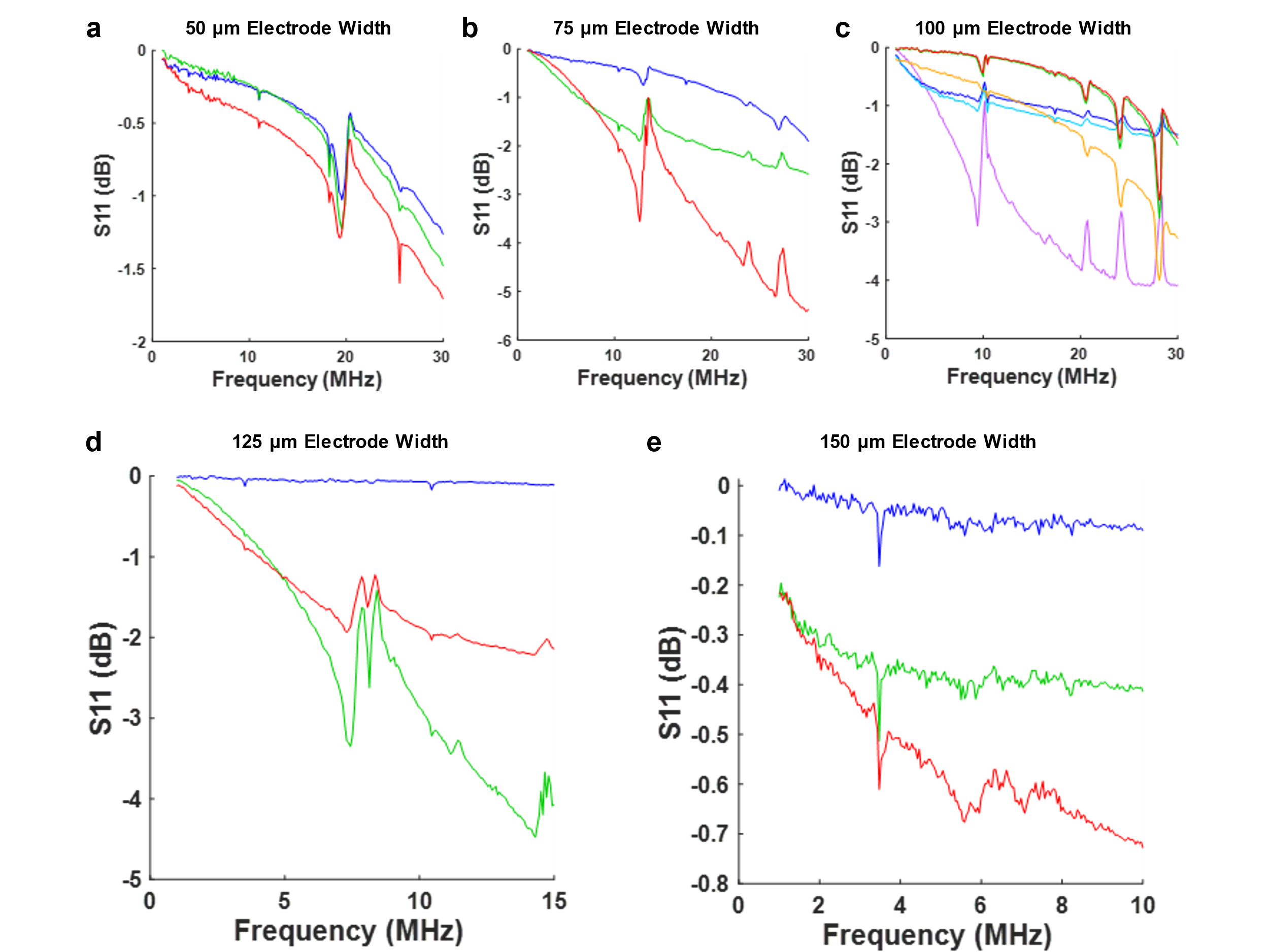


**Supplemental Figure 5.** **S11 network analyzer response of different silver nanowire printed devices** with different electrode widths of (a) 50 μm, (b) 75 μm, (c) 100 μm, (d) 125 μm, and (e) 150 μm. **Sample sizes are 3 different devices, except for the 100 μm silver nanowire devices that have 6.**


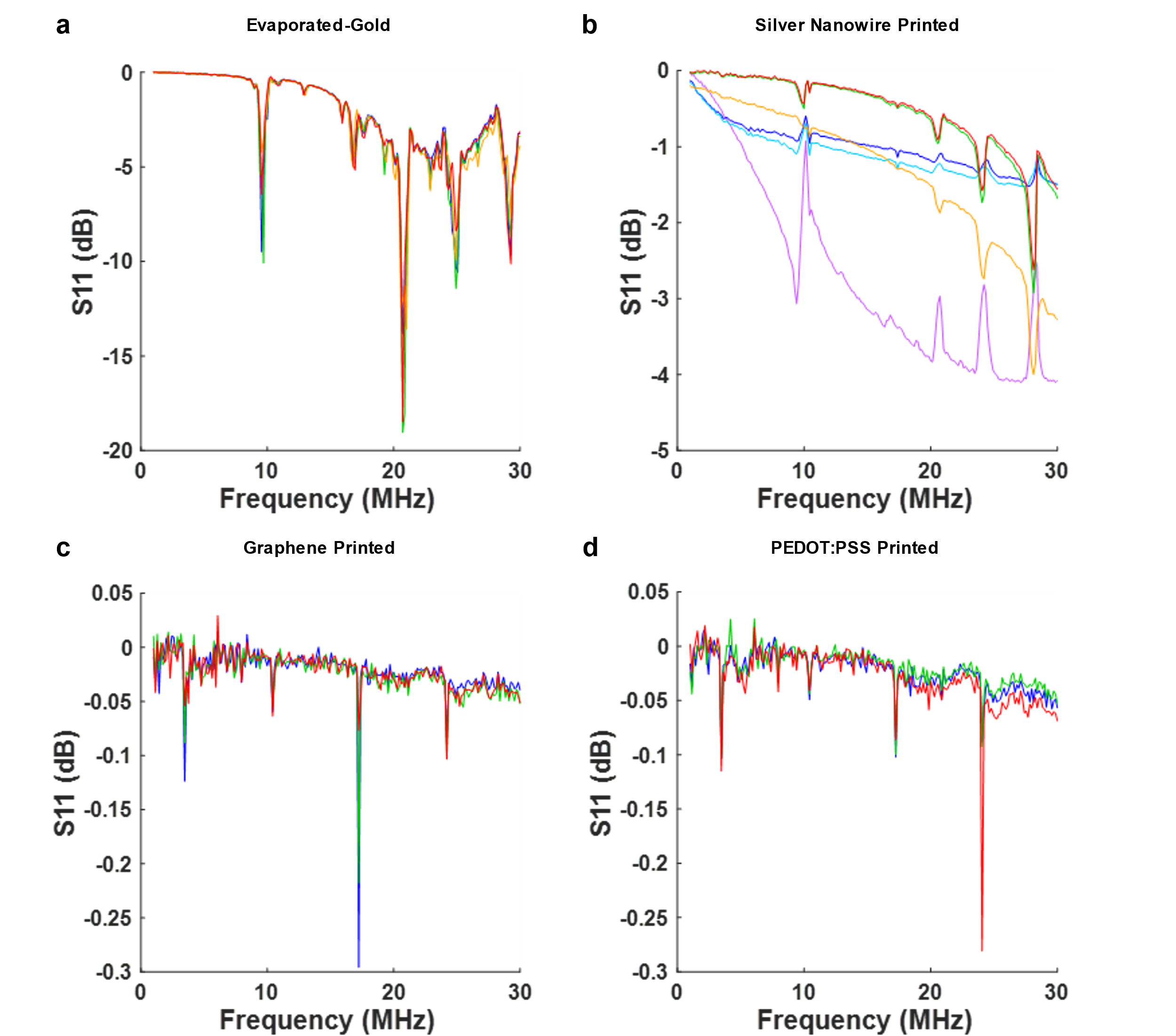


**Supplemental Figure 6.** **S11 network analyzer response of** 100 μm electrode width surface acoustic wave (SAW) microfluidic devices of different materials, including (a) evaporated-gold fabricated device, (b) silver nanowire printed devices, (c) graphene printed devices, and (d) PEDOT:PSS printed devices. **Sample sizes are as follows: evaporated-gold = 4, silver nanowire = 6, graphene = 3, and PEDOT:PSS = 3.**


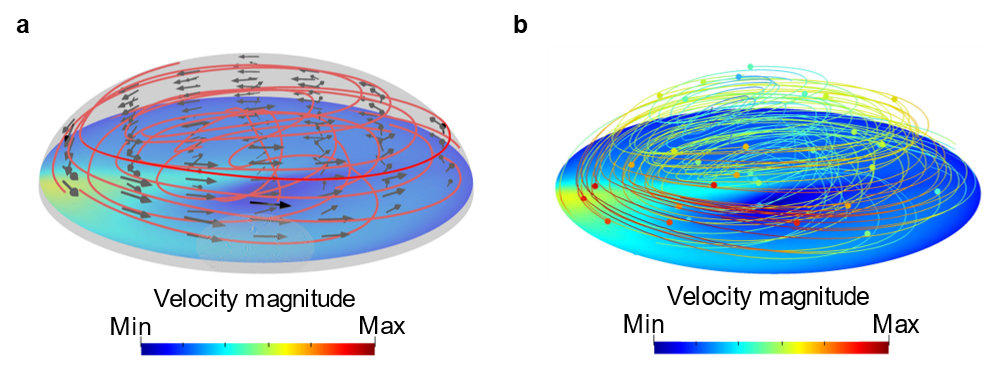


**Supplemental Figure 7.** 3D acoustic simulations within a droplet depicting the (a) **3D acoustic streaming and (b) 3D particle tracing within a droplet.**
